# Editorial: The impact of proteomics on understanding inflammatory and infectious diseases

**DOI:** 10.3389/fimmu.2026.1815841

**Published:** 2026-03-02

**Authors:** Giuseppe G. F. Leite, Victor Corasolla Carregari, Hessel Peters-Sengers, Alexandre Keiji Tashima

**Affiliations:** 1 Division of Infectious Diseases, Department of Medicine, Escola Paulista de Medicina, Universidade Federal de Sao Paulo, São Paulo, Brazil; 2 Department of Laboratory and Hematological Sciences, Unit of Chemistry, Biochemistry and Molecular Biology, “A. Gemelli”, Hospital Foundation IRCCS, Rome, Italy; 3 Center for Infection and Molecular Medicine (CIMM), Amsterdam UMC, University of Amsterdam, Amsterdam, Netherlands; 4 Data Science and Epidemiology, Amsterdam UMC, University of Amsterdam, Amsterdam, Netherlands; 5 Department of Biochemistry, Escola Paulista de Medicina, Universidade Federal de Sao Paulo, São Paulo, Brazil; 6 Laboratory of Mass Spectrometry Applied to Biomolecules, Escola Paulista de Medicina, Universidade Federal de Sao Paulo, São Paulo, Brazil

**Keywords:** biomarker discovery, extracellular vesicles, host response, post-translational modifications (PMTs), precision medicine, shotgun proteomics

Inflammatory and infectious diseases remain major global health challenges, affecting millions of individuals and imposing a sustained burden on healthcare systems. A mechanistic understanding of how host responses interact with pathogens and inflammatory triggers in tissue microenvironments is essential for improving diagnosis, prognosis, and treatment. With the increasing sensitivity, speed, and robustness of modern mass spectrometry platforms, shotgun proteomics has become a central systems-level approach for interrogating these diseases at molecular depth. By quantifying proteins and their modifications, proteomics often offers a more direct window into disease mechanisms than for example transcriptomics.

This Research Topic highlights the transformative power of proteomics and multi-omics in decoding the molecular landscapes of complex human diseases. From acute viral infections and fungal sepsis to chronic autoimmune disorders, the collected articles demonstrate how systematic characterization of the proteome — including post-translational modifications (PTMs) and extracellular vesicle (EV) cargo — can identify potential biomarkers for diagnosis, prognosis, and therapeutic monitoring. The scientific rationale for this Research Topic is grounded in the urgent need for pathogen-agnostic, host response–based strategies to address clinical challenges in which traditional diagnostic tools often fail to provide early or personalized predictive insights.

A first set of studies focuses on the critical need for prognostic biomarkers to guide clinical management of high-mortality infectious diseases. These studies address translational challenges: early discrimination of clinical trajectories and long-term risk. In severe fever with thrombocytopenia syndrome, a proteasome-centered serum proteomic signature identified through machine-learning models prioritized PSMD11 as a robust predictor of adverse outcomes Zhao et al. Similarly, longitudinal plasma profiling in an Ethiopian COVID-19 cohort identified SLAMF1, IL15RA, and IL18 as key markers that not only define acute severity but also predict the risk of post-acute sequelae (Long COVID) Wolday et al. These findings emphasize the transition from broad descriptive proteomics to focused predictive panels adaptable to diverse clinical settings. In addition, these works are especially relevant because longitudinal molecular studies in underrepresented populations remain scarce.

At the diagnostic level, Mnichowska-Polanowska et al., demonstrated that multiplex proximity extension proteomics, a targeted high-throughput approach for measure many proteins simultaneously, can distinguish isolated candidemia from mixed bacterial-fungal sepsis through a compact inflammatory signature (including LAP-TGF-β1, TRANCE, and IL-17C), a distinction with direct implications for antimicrobial stewardship and treatment personalization. Together, these studies exemplify how proteomics coupled to analytical modeling can support risk stratification beyond conventional inflammatory markers.

A second axis of the Topic addresses extracellular vesicles (EVs), which have emerged as a pivotal focus for biomarker discovery and mechanistic insight. In visceral leishmaniasis (VL), a severe parasitic infection caused by Leishmania parasites, the proteomic landscape of plasma-derived EVs revealed 132 human proteins differentially expressed during therapy Torres et al. Notably, serum amyloid A (SAA) emerged as a measurable whole-plasma biomarker for monitoring treatment success. Furthermore, the identification of *Leishmania* proteins within human EV samples provides a novel source of parasite-specific biomarkers for monitoring clinical remission Torres et al.

In the context of COVID-19, circulating EVs from patients with severe disease were enriched in platelet components and molecular signals that exacerbate innate immune-driven inflammation Strippoli et al. This study provided a possible link between EV protein cargo and the enhanced responsiveness of Vδ2 T cells, contributing to the pathogenesis of severe infection Strippoli et al.

The Research Topic also explores the pathophysiological basis of chronic inflammatory diseases. Tissue-level proteomics of intestinal biopsies in ulcerative colitis revealed a dual pathophysiological nature: an exacerbated inflammatory state coupled with substantial metabolic dysfunction, specifically the downregulation of proteins involved in oxidative phosphorylation and the citric acid cycle Wang et al. A similar convergence on fundamental cellular machinery is seen in ankylosing spondylitis, a chronic inflammatory arthritis, where ribosomal proteins (e.g., RPS11, RPS6) in hip-joint tissues were identified as hub nodes linked to osteoblast differentiation and T cell-mediated joint damage Wen et al.

Multi-omics integration is exemplified in rheumatoid arthritis (RA) by Zhang et al., who combined serum proteomics and metabolomics and identified the FBP1/AMPK axis as a key regulator of disease activity. Their integrative analysis also provided a plausible mechanistic explanation for the effects of Qingre Huoxue Decoction: in RA patients and in collagen-induced arthritis mice models, treatment was associated with reduced disease activity, attenuated inflammatory responses, and delayed bone destruction, potentially through FBP1 inhibition and AMPK activation.

Finally, this Topic includes an important conceptual bridge between mechanistic virology and therapeutic innovation. In a comprehensive review, Li et al. synthesize how proteome-wide characterization of post-translational modifications (PTMs) illuminates host–virus interactions and identifies putative antiviral drug targets. The broader implication is straightforward: if infection outcomes are shaped by dynamic protein states rather than static abundance alone, then PTM-aware proteomics becomes indispensable for next-generation host-directed therapies.

Collectively, the studies in this Topic support a cautious but constructive conclusion. Proteomics is most informative when embedded in translational designs that combine deep clinical phenotyping, longitudinal sampling, and orthogonal validation, because biological context and temporal structure determine whether molecular signals are interpretable. Compact protein signatures are promising for risk stratification and monitoring, but clinical utility requires more than statistical discrimination: signatures should be benchmarked against standard-of-care markers, developed with explicit control of confounding (including co-infections and treatment effects), and externally validated across independent populations, analytical platforms, and care settings. Mechanistically, pathway- and network-level analyses are generally more robust than isolated marker reporting, especially when integrated with PTM- and EV-resolved layers and supported by functional validation.

At the same time, this body of work also clarifies current bottlenecks: relatively small discovery cohorts in several settings, limited harmonization of sampling timepoints, and the need for deeper multi-center validation before routine clinical implementation. Addressing these gaps will require standardized workflows, transparent analytical pipelines, and prospective studies designed for decision-support endpoints.

Despite these challenges, the direction is clear. Proteomics is reshaping how we define inflammatory and infectious diseases: not as static labels, but as dynamic molecular trajectories that can be measured, modeled, and eventually modulated. We thank all authors for their contributions and hope this Research Topic helps accelerate the transition from molecular insight to precision care.

